# Acute pancreatitis as the initial manifestation of acute myeloid leukemia: a case report and literature review

**DOI:** 10.3389/fmed.2026.1924463

**Published:** 2026-07-31

**Authors:** Dandan Chen, XianYi Lin, Xiaohong Cai, Xiangqi Huang, Churong Lin, Ruijuan Wen, Xiangzhong Zhang, Xudong Li, Lingling Liu

**Affiliations:** 1 Department of Infectious Diseases, The Third Affiliated Hospital of Sun Yat-sen University, Guangzhou, Guangdong, China; 2 Department of Gastroenterology, The Third Affiliated Hospital of Sun Yat-sen University, Guangzhou, Guangdong, China; 3 Department of Hematology, The Third Affiliated Hospital of Sun Yat-sen University, Guangzhou, Guangdong, China; 4 Department of Pathology, The Third Affiliated Hospital of Sun Yat-sen University, Guangzhou, Guangdong, China; 5 Department of Radiology, The Third Affiliated Hospital of Sun Yat-sen University, Guangzhou, Guangdong, China

**Keywords:** acute myeloid leukemia, acute pancreatitis, case report, extramedullary infiltration, initial manifestation

## Abstract

**Background:**

Acute myeloid leukemia (AML) presenting with acute pancreatitis (AP) as the initial symptom is extremely rare. Severe AP (SAP) has a significant death rate and advances quickly. Therefore, it is crucial to identify the predisposing factors in the early stages of SAP, assess the condition, establish the prognosis, develop treatment plans and prevent a recurrence. Here, we describe a case of AP associated with AML infiltration involving the pancreatic segment of the common bile duct.

**Case summary:**

A 34-year-old man arrived to the hospital complaining of persistent abdominal pain. Blood analysis presented elevated serum lipase levels, leukocytosis, and severe thrombocytopenia. Computed tomography examination of the abdomen revealed necrotizing pancreatitis, accompanied by significant stenosis of the pancreatic segment of the common bile duct due to extrinsic compression, and moderate dilatation of the proximal bile duct and intrahepatic biliary ducts. The patient had no typical etiologies of acute pancreatitis except a history of type 2 diabetes mellitus, which alone could not account for the significant thrombocytopenia observed concurrently. Finally, the bone marrow aspirate and smear examination identified the acute myeloid leukemia (AML) which was considered as the primary cause of pancreatitis.

**Conclusion:**

Acute leukemia, particularly AML, is an uncommon cause of AP via direct pancreatic infiltration by leukemic cells. Although AP rarely presents as an extramedullary manifestation in AML patients, it should be considered in the etiological workup of AP. Early recognition and targeted etiological management can optimize clinical outcomes and prevent ineffective treatment.

## Introduction

Acute pancreatitis (AP) is a common gastrointestinal emergency, most frequently caused by gallstone disease and excessive alcohol consumption, which together account for approximately 70–80% of cases (1). Other known etiologies include hypertriglyceridemia, hypercalcemia, drug-induced pancreatitis, trauma, and iatrogenic factors. Despite thorough diagnostic evaluation, approximately 10–20% of cases remain classified as idiopathic pancreatitis, for which uncommon and often overlooked causes should be carefully considered (2).

Among these rare etiologies, pancreatic or biliary system infiltration by hematological malignancies is extremely uncommon yet clinically important. Acute myeloid leukemia (AML) is a clonal disorder originating from hematopoietic precursor cells that primarily involves the bone marrow and peripheral blood and extramedullary involvement may also occur (3). However, pancreatic and biliary tract involvement by leukemic cells is particularly rare and may cause ductal obstruction, cholestasis, and secondary pancreatic inflammation, thereby mimicking the clinical presentation of gallstone pancreatitis or pancreatic malignancy. Consequently, diagnosis is often delayed, presenting substantial challenges in clinical practice. Recent case reports have highlighted the importance of including hematologic malignancies in the differential diagnosis of unexplained acute pancreatitis or obstructive jaundice, especially when conventional etiologies have been ruled out.

We herein report a rare case of AML that initially presented with acute pancreatitis. The patient subsequently developed progressive obstructive jaundice secondary to leukemic infiltration of the common bile duct, and biliary obstruction was finally relieved by endoscopic retrograde cholangiopancreatography (ERCP) combined AML-targeted chemotherapy. This case aims to heighten clinicians’ awareness of this unusual manifestation and underscore the importance of early diagnosis and multidisciplinary evaluation.

## Case presentation

A 34-year-old male was admitted to the Gastroenterology Department of our hospital with a 7-day history of abdominal pain accompanied by vomiting and decreased appetite but without fever. He denied early fatigability, and neither he nor his family members reported significant weight loss prior to admission. There was no history of gastrointestinal bleeding or other significant blood loss. Magnetic resonance cholangiopancreatography (MRCP) findings were suggestive of hemorrhagic necrotizing pancreatitis. Symptoms began 7 days prior to admission with persistent epigastric pain, which gradually progressed to generalized abdominal pain. Physical examination revealed tenderness in the upper abdomen. This patient had a history of type 2 diabetes mellitus and hyperuricemia, but no hypertension, hyperlipidemia, or coronary artery disease. There was no history of alcohol consumption or smoking. The patient denied recent trauma or exposure to chemotherapy. There was no significant family history.

**Figure 1:**
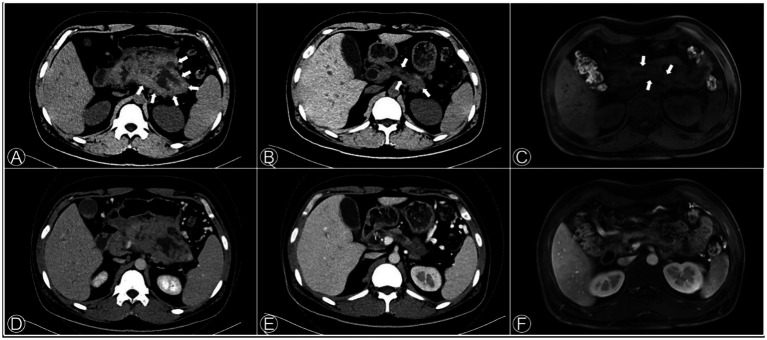
Imaging of infiltration of the pancreas and acute pancreatitis. **(A–C)** Non-contrast images. **(D–F)** Corresponding portal venous phase contrast-enhanced images at identical scan levels **(A–D, B–E, C–F)**. **(A,D)** Abdominal computed tomography (CT) showed pancreatic necrosis with diffuse peripancreatic inflammatory exudation (white arrow). **(B,E)** Abdominal contrast-enhanced CT showed a reduction in pancreatic size compared with prior imaging, along with decreased peripancreatic inflammatory exudation (white arrow). **(C,F)** Upper abdominal magnetic resonance imaging (MRI) showed a reduction in pancreatic enlargement and decreased peripancreatic inflammatory exudation compared with the previous CT examination (white arrow).

Initial laboratory testing upon admission indicated a white blood cell (WBC) count of 20.59 × 10^9^/L (reference range: 3.5–9.5 × 10^9^/L), with a monocyte percentage of 40.70%, a low platelet count of 85 × 10^9^/L (reference range: 100–350 × 10^9^/L), and a hemoglobin level of 137 g/L (reference range: 130–175 g/L). C-reactive protein was 64.42 mg/L (reference range: 0–6.0 mg/dL). Serum lipase was mildly increased (63 U/L, reference range: 0–60 U/L), total bilirubin was elevated at 71.05 μmol/L (reference range: 0–26 μmol/L), and serum calcium was 2.10 mmol/L (reference range: 2.11–2.52 U/L). Serum amylase and triglyceride levels were within normal limits. Contrast-enhanced whole-abdomen computed tomography (CT) revealed the following: (1) Pancreatic findings suggest necrotizing pancreatitis, with multiple necrotic encapsulations forming throughout the pancreas, accompanied by diffuse inflammatory exudate. Involvement of the gastric wall and duodenum is suspected. (2) Irregular dilation of portions of the pancreatic ducts is noted; caution is warranted for the possibility of an acute exacerbation of chronic pancreatitis (Figures 1A,D).

**Figure 2:**
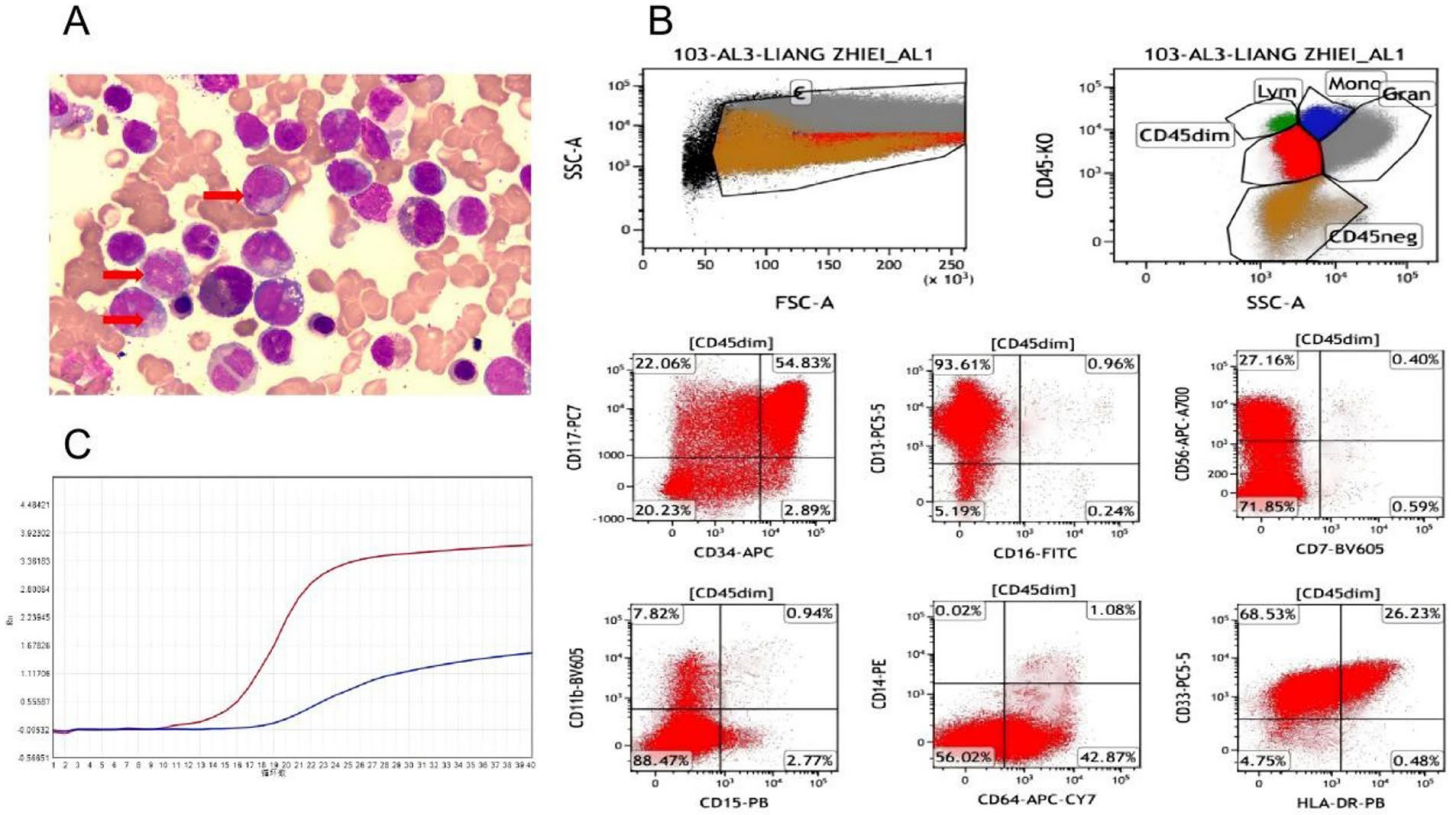
Bone marrow smear examination, fusion gene analysis, and mutation testing. **(A)** Bone marrow cell morphology revealed increased blast cells. **(B)** Flow cytometry analysis showed positive expression of CD34, CD117, HLA-DR, CD56, CD64, CD33, and CD13. **(C)** The blue horizontal line represents the threshold. The blue curve indicates the internal control gene in the Cy5 channel, while the red curve represents CBFβ-MYH11 detected in the FAM channel for this patient.

Given the patient’s leukocytosis with concurrent monocytosis and thrombocytopenia, gastroenterologists consulted the hematology department, with a recommendation for bone marrow aspiration to rule out hematological malignancy. Bone marrow smear demonstrated abnormal proliferation of primitive and immature cells accounting for 53%, consistent with AML M4-EO (eosinophilia) subtype (Figure 2A). Flow cytometry analysis showed that CD45dim cells constitute approximately 28.5% of total nucleated cells in the submitted specimen. Their immunophenotype was as follows: CD45dim, CD38dim, partially positive for CD34, CD117, HLA-DR, CD56, CD64, and positive for CD33 and CD13; negative for CD11b, CD15, CD16, CD14, CD7, CD3, CD2, CD5, CD4, CD8, CD19, CD10, CD20, CD22, C36, and CD71 (Figure 2B). The leukemia fusion gene CBFβ-MYH11 was positive (Figure 2C). Cytogenetic analysis of the bone marrow showed a 46, XY karyotype. Gene mutation testing for myeloid malignancies identified NRAS (c.181C > A; p. Q61K) and CBL (c.1096G > A; p. E366K) mutation. Thus, based on the clinical manifestations, medical history, and laboratory results, the final diagnosis was AML predominantly presenting with severe acute pancreatitis (SAP) secondary to extramedullary infiltration and was stratified into the favorable-risk group.

Following transfer to the Hematology Department, the patient was initiated on hydroxyurea to reduce the white blood cell count. Concurrent supportive care included infection control, gastric protection, liver function support to alleviate jaundice, and blood glucose management. Then the symptoms of abdominal pain were alleviated and the inflammation was reduced. Nevertheless, the patient’s obstructive jaundice persisted and progressively worsened. ERCP and endoscopic retrograde biliary drainage (ERBD) were performed. Subsequently, the patient received induction chemotherapy with idarubicin and cytarabine (IA regimen). After induction chemotherapy, the patient achieved complete remission (CR) and minimal residual disease (MRD) negativity of AML, and the AP was completely resolved. Subsequently, the patient received two cycles of consolidation chemotherapy: the IA regimen and high-dose cytarabine (HD-Ara-C) regimen, respectively. At the second follow-up visit after his first discharge, by which time he had completed two cycles of chemotherapy, he developed complications including febrile neutropenia and subarachnoid hemorrhage secondary to severe thrombocytopenia. The laboratory tests revealed severe pancytopenia, with a white blood cell count of 0.11 × 10^9^/L (reference range: 3.5–9.5 × 10^9^/L), an absolute neutrophil count of 0 × 10^9^/L (reference range: 1.8–6.3 × 10^9^/L), a hemoglobin level of 44 g/L (reference range: 130–175 g/L), and a platelet count of 4 × 10^9^/L (reference range: 100–350 × 10^9^/L). The C-reactive protein level was 159.48 mg/L (reference range: 0–6.0 mg/dL), and the total bilirubin level increased to 32.38 μmol/L (reference range: 0–26 μmol/L). The serum amylase level was 8 U/L (reference range: 35–135 U/L), while the serum lipase and triglyceride levels remained within the normal range. After then, the patient achieved continuous MRD negativity for AML. Abdominal contrast-enhanced CT performed two months after ERCP revealed significant improvement of pancreatitis and biliary obstruction (Figures 1B,E), and the biliary stent was thereafter removed following resolution of the pancreatic lesions and obstructive jaundice. At a subsequent 2-month follow-up, upper abdominal enhanced magnetic resonance imaging (MRI) showed improvement of pancreatitis and decreased common bile duct dilatation (Figures 1C,F). Then, the patient underwent autologous hematopoietic stem cell transplantation (HSCT) and is currently under follow-up after the transplantation. The patient has been followed up for more than four months since autologous stem cell infusion (March 7, 2026). During follow-up, no further episodes of AP occurred. Bone marrow examinations were conducted at the 1st, 2nd and 3rd month post-transplantation. Results of bone marrow smear, flow cytometry and previous pathogenic gene testing remained persistently negative. The patient experienced transient poor platelet engraftment after transplantation. After treatment with platelet-raising medications, the peripheral blood cell counts returned to normal completely.

## Discussion

Acute leukemia-related pancreatitis has been predominantly reported in pediatric patients with acute lymphoblastic leukemia (ALL), with most cases attributed to the asparaginase-based chemotherapy. Pancreatitis has also been documented in AML patients receiving cytarabine (4, 5), all-trans retinoic acid (6–8), and various combination chemotherapy regimens (9). Chemotherapy-induced pancreatitis is generally mild and often resolves upon drug discontinuation. Additionally, pancreatitis has been associated to bone marrow transplantation (10–12). Hypercalcemia caused a small number of AP patients with adult T-cell leukemia (13). In contrast, AP as the initial presenting manifestation of acute leukemia at the diagnosis is extremely rare. Only 32 cases of acute leukemia involving pancreas reported in the literature to date (Table 1). A review of the previously reported 32 cases demonstrates several shared clinical characteristics of pancreatic lesion associated with acute leukemia. Among these 32 cases, 18 were diagnosed with ALL, while 14 were AML. Extramedullary infiltration may occur at the time of initial leukemia diagnosis or emerge as an important indicator of relapse. In this retrospective analysis, six cases involved relapse, whereas AP constituted the initial manifestation of leukemia in all other cases. According to the literature, approximately 25–50% of patients with acute leukemia experience relapse following hematopoietic stem cell transplantation, and among these relapses, about 15–23% involve extramedullary sites (14). This suggests that leukemic activity rather than primary pancreatic disease may be the cause of recurrent or atypical pancreatitis. Compared with patients in whom leukemia presented initially, those with pancreatic involvement at relapse exhibited more prominent clinical manifestations, including significant abdominal pain, nausea, and vomiting consistent with AP, or marked jaundice with elevated bilirubin levels. Moreover, AP was caused by pancreatic myeloid sarcoma in seven cases (11, 15–20), whereas in the majority of the remaining cases it was attributed to leukemic infiltration of the pancreas. AP was first categorized as idiopathic pancreatitis accompanied by unexplained cytopenias or aberrant hematologic results because the majority of patients had none of the traditional causes of pancreatitis, such as gallstones, alcohol consumption, or hypertriglyceridemia. Imaging examinations showed that extramedullary myeloid sarcoma, which often involved the pancreatic head or pancreatobiliary system, or leukemic infiltration were the main causes of pancreatic involvement. As a result, these lesions were often misdiagnosed as autoimmune pancreatitis (11, 12, 21) or pancreatic malignancy (15, 16, 19). AML subtypes with chromosome 16 abnormalities (20) and monocytic-lineage subtypes (FAB M4/M5) (16, 17, 22, 23), both of which are known to show a higher propensity for extramedullary infiltration, are more likely to affect the pancreas. Clinical outcomes among reported cases were highly heterogeneous. Most of the patients had short- to intermediate-term survival, especially when systemic treatment was started as soon as acute leukemia was identified. Patients with pancreatic myeloid sarcoma, on the other hand, had worse results with higher rates of relapse and mortality, particularly those that developed after stem cell transplantation (11, 16, 19). Overall, leukemia status at final follow-up varied from total remission to disease progression or relapse, suggesting that pancreatic involvement is more indicative of systemic leukemia behavior than of isolated pancreatic pathology.

**Table 1:** Cases of acute leukemia involving pancreas.

References	Age/sex	Leukemia type	AP is the initial symptom of leukemia	Symptoms and laboratory/imaging abnormalities associated with acute pancreatitis	Recurrent episodes (≥ 2) of acute pancreatitis	Cause of pancreatitis	Treatment for leukemia	Outcome
Our case	34 y/Male	AML (M4)	Yes	Epigastric pain, vomiting, increased serum pancreatic enzymes, multiple necrotic encapsulations in the pancreas accompanied by diffuse inflammatory exudate by CT	No	Suspected infiltration of AML cells to the pancreas	Intensive chemotherapy, HSCT	Alive, 8 months after AML diagnosis
Sumitani et al. (21)	33 y/Male	AML	Yes	Epigastric pain, increased serum pancreatic enzymes, diffuse swelling of the pancreas and diffuse beaded dilatation of the main pancreatic duct by CT and MRCP	Yes	Suspected infiltration of AML cells to the pancreas	Intensive chemotherapy	Alive, 3 years after AML diagnosis
Ozeki et al. (12)	30 y/Male	AML (M2)	No	Back pain, increased serum pancreatic enzymes, swelling of the pancreatic body by CT	Yes	Documented infiltration of AML cells to the pancreas	Intensive chemotherapy, HSCT	Dead, 2 years and 9 months after AML diagnosis
Kamada et al. (11)	37 y/Female	AML	No	Epigastric pain, increased serum pancreatic enzymes, diffuse swelling of the pancreas and a mass in the pancreatic head by CT	Yes	Suspected extramedullary sarcoma of the pancreatic head	Intensive chemotherapy, HSCT	Dead, 6 years and 4 months after AML diagnosis
Clausen et al. (15)	19 y/Female	AML	No	Epigastric pain, nausea and vomiting, increased serum pancreatic enzymes, and a mass in the pancreatic head by CT, PET-CT and EUS	No	Extramedullary sarcoma of the pancreas head (EUS-FNA)	Intensive chemotherapy, HSCT	Alive, 2 years after AML diagnosis
Schäfer et al. (16)	75 y/Female	AML (M4eo)	Yes	Epigastric pain, increased serum pancreatic enzymes, and a diffusely infiltrating mass in the pancreatic head by MRI	No	Suspected extramedullary sarcoma of the pancreatic head and diffusely infiltrating AML cells	Intensive chemotherapy	Dead, 7 months after attaining CR
Norsworthy et al. (28)	39 y/Male	AML	Yes	Abdominal pain radiating to the back, diffusely abnormal pancreas, and enlarged peripancreatic lymph nodes by EUS	Yes	Suspected infiltration of AML cells to the pancreas	Intensive chemotherapy, HSCT	Dead, 11 months after AML diagnosis
Tabriz et al. (17)	47 y/Male	AML (M5A)	No	Epigastric pain, increased serum pancreatic enzymes, and a mass in the pancreatic head with edematous pancreas from head to tail by CT and EUS	Yes	Diffusely infiltrating extramedullary sarcoma of the pancreas head (laparotomy)	Intensive chemotherapy, HSCT	Alive at time of report
Ishii et al. (18)	19 y/Male	AML	No	Epigastric pain, increased serum pancreatic enzymes, and a diffusely infiltrating mass in the pancreatic head by CT, MRI and PET-CT	No	Documented infiltration of AML cells to the pancreas (EUS-FNA)	Intensive chemotherapy	Alive at time of report
Zhu et al. (24)	36y/Male	AML	Yes	Epigastric pain, increased serum pancreatic enzymes, and a diffusely infiltrating mass in the pancreatic tail by CT, EUS and PET-CT	Yes	Documented infiltration of AML cells to the pancreas (percutaneous FNA)	Intensive chemotherapy	Alive, 1 year and 7 months after AML diagnosis
Messager et al. (19)	19 y/Female	AML	Yes	Epigastric pain, increased serum pancreatic enzymes, and a mass in the pancreatic head by MRI	Yes	Extramedullary sarcoma of the pancreas head (laparotomy)	Intensive chemotherapy, HSCT	Dead, 1 year and 8 months after AML diagnosis
Fukushima et al. (20)	35 y/Male	AML (M1)	Yes	Epigastric pain, increased serum pancreatic enzymes, diffusely infiltrating mass in the pancreatic head by CT, MRI, MRCP and ERCP	Yes	Extramedullary sarcoma of the pancreas head (EUS-FNA)	Intensive chemotherapy	Alive, 2 months after AML diagnosis
Yang et al. (29)	61 y/Male	AML (M2)	Yes	Epigastric pain, increased serum pancreatic enzymes, pancreatitis with diffuse edema by CT	No	Suspected infiltration of AML cells to the pancreas	Intensive chemotherapy	Alive, 1 years after AML diagnosis
Y et al. (22)	32 y/Male	AML (M5)	Yes	Back pain, abdominal pain, increased serum, pancreatic enzymes, enlarged pancreas by CT	No	N. A	Hydroxyurea tablet	Dead at time of report
Feng et al. (23)	61 y/Male	AML (M5)	Yes	Epigastric pain, increased serum pancreatic enzymes, enlarged and indistinct pancreas by CT	No	N. A	Chemotherapy	Alive at time of report
Zhou et al. (30)	54 y/Male	ALL	Yes	Epigastric pain, increased serum pancreatic enzymes, slight swelling of the pancreas by CT	No	N. A	Chemotherapy	Alive at time of report
Yadav et al. (31)	25 y/Female	ALL	Yes	Fever and epigastric pain, increased serum pancreatic enzymes, enlarged pancreas and hypodense lesions in pancreas by CT	No	N. A	Antibiotics, symptomatic and supportive care, no chemotherapy	Dead at time of report
Pamuk et al. (32)	52 y/Male	ALL	Yes	Abdominal pain, nausea and vomiting, increased serum pancreatic enzymes, enlarged pancreas by CT	No	Documented infiltration of ALL cells to the pancreas	Chemotherapy	Dead, 6 months after ALL diagnosis
Hanbali et al. (33)	42 y/Male	ALL	Yes	Epigastric pain, increased serum pancreatic enzymes, fat stranding and haziness around the pancreas by CT	No	N. A	Chemotherapy	Dead at time of report
Mori et al. (34)	50 y/Female	ALL	Yes	Backache and appetite loss, increased serum pancreatic enzymes, diffusely enlarged pancreas and dilation of the main pancreatic duct by CT	No	Documented infiltration of ALL cells to the pancreas (EUS-FNB)	Chemotherapy	Dead, 4 months after ALL diagnosis
Sato et al. (35)	5 y/Male	ALL	No	Abdominal pain, increased serum pancreatic enzymes, swelling of the pancreas by CT	Yes	N. A	Intensive chemotherapy	Alive at time of report
Wang et al. (36)	12 y/Male	ALL	No	Epigastric pain and scleral icterus, increased serum pancreatic enzymes, diffusely enlarged pancreas and intra- and extrahepatic biliary dilatation by US and MRI	Yes	Documented infiltration of ALL cells to the pancreas (US-guided pancreas biopsy)	Intensive chemotherapy, HSCT	Dead, 3 year and 1 months after ALL diagnosis
Wang et al. (37)	39 y/Female	ALL	Yes	Recurrent fever, multiple hypoattenuating mass lesions in pancreas by CT and multiple lesions by MRI	No	Documented infiltration of ALL cells to the pancreas (EUS-FAN)	Intensive chemotherapy, HSCT	Dead, 12 months after ALL diagnosis
Guruprasad et al. (38)	10 y/Male	ALL	Yes	Fever, leg pains, increased serum, pancreatic enzymes, diffusely enlarged pancreas by US and CT	No	Documented infiltration of ALL cells to the pancreas (EUS-FANC)	Chemotherapy	Alive at time of report
Ramanathan et al. (39)	28 y/Male	ALL	Yes	Fever and black stools, multiple tiny hypodense lesions in pancreas by CT	No	Suspected infiltration of ALL cells to the pancreas	No chemotherapy	Dead at time of report
Collado et al. (40)	3 m/Female	ALL	Yes	Vomiting, enlarged pancreas with multiple hypoechogenic lesions by US	No	N.A	Chemotherapy	Alive at time of report
Malbora et al. (41)	4 y/Male	ALL	Yes	Fever, increased serum, pancreatic enzymes, enlarged pancreas and diffuse hypodense lesions in pancreas by CT	No	Documented infiltration of ALL cells to the pancreas	Chemotherapy	Alive, 11 months after ALL diagnosis
Ikawa et al. (42)	11 y/Female	ALL	Yes	Fever and lumbago, enlarged pancreas and diffuse hypodense lesions in pancreas by CT	No	N. A	Chemotherapy	Alive at time of report
Abdurrahman (43)	20 m/Male	ALL	Yes	Jaundice and abdominal pain, Enlarged pancreas, dilated bile ducts by CT	No	Documented infiltration of ALL cells to the pancreas	Chemotherapy	Alive at time of report
Daniel et al. (44)	39 y/Male	ALL	Yes	Epigastric pain radiating to the back and jaundice, enlarged pancreas with dilatation of the common bile duct and intrahepatic biliary tree by CT	No	N. A	Chemotherapy	Alive at time of report
Rausch et al. (45)	10 w/Female	ALL	Yes	Jaundice, enlarged pancreas and intra- and extrahepatic biliary dilatation by US	No	N. A	chemotherapy	Dead at time of report
Deng et al. (46)	60 y/Male	ALL	No	Jaundice, enlarged pancreatic head, dilation of the intra- and extrahepatic bile ducts and the pancreatic duct by CT	No	N. A	Intensive chemotherapy	Dead, 2 year and 6 months after ALL diagnosis
Su et al. (47)	58 y/Female	ALL	Yes	Epigastric pain, increased serum, enlarged head of pancreas by CT	No	Suspected infiltration of ALL cells to the pancreas	Intensive chemotherapy	Alive at time of report

Thirty-two cases of acute leukemia involving pancreas are listed. AML subtype is shown for each of the cases according to the French-American-British (FAB) classification. N.A. not available, HSCT hematopoietic stem cell transplantation, PET-CT positron emission topography and computed tomography, EUS endoscopic ultrasound, FNA fine needle aspiration, FNB fine needle biopsy, US ultrasound, MRI magnetic resonance imaging.

Extramedullary leukemia can affect any organ system. Isolated organ damage or noticeable localized manifestations are frequently the initial indications. Leukemia-related AP is relatively uncommon in AML, and its underling pathogenesis remains unclear. Direct infiltration of leukemic cells into the pancreatic parenchyma has been implicated in rare AML cases presenting with AP (12, 18, 24). Studies have suggested that microenvironmental factors, including the CXCR4/CXCL12 axis and matrix metalloproteinases, may promote tumor cell migration, thereby facilitating tissue invasion and destruction (25). In addition, clinical cases of extramedullary sarcoma involving the pancreas further illustrate the capacity of myeloid blasts to colonize and disrupt normal pancreatic architecture (15, 17, 19, 20). Broader AML research identifies elevated levels of pro-inflammatory mediators such as TNF-*α* and IL-6 within leukemic microenvironments, suggesting that local inflammatory signaling may contribute to acinar injury (26). Regardless of whether serum amylase was elevated, AP should be considered in acute leukemia patients experiencing acute and persistent epigastric pain. Early diagnosis and intervention can improve the prognosis of affected patient. Otherwise, it may rapidly progress to SAP, resulting in organ failure and even mortality. Given its insidious presentation and severe clinical consequences, clinicians should maintain heightened awareness of this complication.

This result is still up for debate, even though extramedullary infiltration in AML is thought to be a poor prognostic predictor (27). The patient we reported was stratified into the low-risk prognostic group and achieved favorable survival following timely diagnosis and treatment. The presentation of AP as the initial manifestation did not appear to exert a severe adverse impact on prognosis.

## Conclusion

AP may present as the initial manifestation of AML, posing a substantial diagnostic challenge. Clinicians should maintain a high index of suspicion for any underlying hematologic malignancies in patients with idiopathic acute pancreatitis. For those with unexplained leukocytosis, anemia, or thrombocytopenia, bone marrow examination are strongly advised. Careful assessment of routine blood parameters is crucial, as early identification of hematologic abnormalities can enable timely diagnosis and treatment.

## Data Availability

The original contributions presented in the study are included in the article/supplementary material, further inquiries can be directed to the corresponding authors.
